# Mimickers of chronic thromboembolic pulmonary hypertension on imaging tests: a review

**DOI:** 10.1177/2045894019882620

**Published:** 2020-03-26

**Authors:** Shraddha Narechania, Rahul Renapurkar, Gustavo A. Heresi

**Affiliations:** 1 Respiratory Institute, Cleveland Clinic, Cleveland, OH, USA; 2 Department of Diagnostic Radiology, Cleveland Clinic, Cleveland, OH, USA

**Keywords:** mimicker, thromboembolism, thrombosis, pulmonary hypertension, chronic thromboembolic pulmonary hypertension

## Abstract

Chronic thromboembolic pulmonary hypertension (CTEPH) is caused by mechanical obstruction of large pulmonary arteries secondary to one or more episodes of pulmonary embolism. Ventilation perfusion scan is the recommended initial screening test for this condition and typically shows multiple large mismatched perfusion defects. However, not all patients with an abnormal ventilation perfusion scan have CTEPH since there are other conditions that be associated with a positive ventilation perfusion scan. These conditions include in situ thrombosis, pulmonary artery sarcoma, fibrosing mediastinitis, pulmonary vasculitis and sarcoidosis, among others. Although these conditions cannot be distinguished from CTEPH using a ventilation perfusion scan, they have certain characteristic radiological features that can be demonstrated on other imaging techniques such as computed tomography scan and can help in differentiation of these conditions. In this review, we have summarized some key clinical and radiological features that can help differentiate CTEPH from the CTEPH mimics.

## Introduction

Chronic thromboembolic pulmonary hypertension (CTEPH) is caused by mechanical obstruction of major pulmonary arteries due to one or recurrent pulmonary thromboemboli, which is followed by organization of the thrombus and small vessel arteriopathy.^1,2^ It is an important reversible cause of pulmonary hypertension (PH), which is frequently underdiagnosed.^1^ CTEPH is classified as group 4 PH by the World Symposium of PH.^3^ It is the only form of PH that is potentially curable. Early and accurate recognition is therefore vital to prevent progression and complications like progressive PH, right heart failure and death.^1^ Delayed diagnosis may be associated with a worse prognosis, higher perioperative mortality, and inoperable disease stages.^4^

The clinical features of CTEPH are non specific and similar to those seen in other forms of PH. Ventilation perfusion (VQ) scanning is the initial screening test of choice to detect CTEPH. Some of the key advantages include, ease of availability and simplicity of interpretation.^5,6^ A normal V/Q scan virtually excludes CTEPH. At least one, and more commonly multiple mismatched perfusion defects are seen in patients with CTEPH.^5,7^ An abnormal VQ scan should be followed with pulmonary angiography (via computed tomography, magnetic resonance imaging and/or conventional digital subtraction angiography) to confirm the presence of CTEPH. Altered resolution of thrombus in the pulmonary arteries appears as webs, bands, partial recanalization, stenosis, pouch defects and retraction of the vessel with partial or total occlusion.^8^ However, one need to be cognizant of other causes of PH that can mimic CTEPH due to similarities on imaging tests.^5^

In this article, we highlight some important clinical as well as radiologic characteristics that can help distinguish CTEPH from other processes that can act as mimickers of this disease (Table 1).

**Table 1.** Salient features of conditions mimicking CTEPH.

	CTEPH Mimic	Similarities on imaging tests	Dissimilarities on imaging tests	Conditions in which the mimic should certainly be considered
1	In situ thrombosis in congenital heart disease	Mosaic attenuation seen but less pronounced Bronchial collaterals seen but less common Central pulmonary artery thrombus	Gross disproportionate enlargement of the central pulmonary arteries Absence of other vascular signs, such as vascular webs, vessel beading or paucity of peripheral arteries, beyond the central pulmonary arteries VQ scan normal or mottled appearance	Patients with congenital heart disease Eisenmenger's physiology
2	Pulmonary artery sarcoma	Perfusion defects on VQ scan Pulmonary arterial intraluminal filling defect	Complete vessel occlusion Expansile mass Extraluminal extension Involvement of pulmonary valve and RVOT Heterogenous attenuation PET avidity Delayed enhancement on CTA and MRI	Increase in thrombus size despite anticoagulation Pulmonary nodules Movement with cardiac cycle
3	Pulmonary vein involvement: a) Fibrosing mediastinitis b) Pulmonary vein stenosis secondary to ablation for atrial fibrillation	a) Mismatched perfusion defects on VQ scan b) Mismatched perfusion defects on VQ scan	a) Mediastinal and hilar soft tissue masses Mediastinal calcification Central pulmonary artery stenosis Pulmonary venous stenosis Airway narrowing Mediastinal lymphadenopathy b) Filling defects in the pulmonary veins Interlobulat septal thickening, pleural effusions, Kerley B lines on CXR	a) Prior history of histoplasmosis History of recurrent pulmonary infections b) Prior history of ablation for atrial fibrillation
4	PVOD	Mosaic attenuation pattern in lung fields	VQ scan normal in most cases Smooth interlobular septal thickening Ground glass opacities Mediastinal lymphadenopathy	Development of pulmonary edema following pulmonary vasodilator therapy
5	Large vessel vasculitis	Collateralization of vessels	Abnormal aortic contour and mural thickening Pulmonary aneurysms Transmural arterial calcification Absent intraluminal thrombus PET avidity	Asian women (Takayasu's arteritis) Visual defects, limb claudication, fever Concomitant presence of stroke, myocardial ischemia
6	Sarcoidosis	Mismatched perfusion defects on a VQ scan Arterial webbing Intimal irregularities Vascular narrowing with post stenotic dilatation	Absence of filling defects in the pulmonary arteries Parenchymal involvement Upper lobe predominance Mediastinal lymphadenopathy causing extrinsic compression	Evidence of sarcoidosis in extra pulmonary organs
7	Malignancy	VQ scan with multiple mismatched perfusion defects	Lymphangitic carcinomatosis PET avidity in some cancers	Significant unintentional weight loss Current or prior malignancy Evidence of metastatic disease Mediastinal lymphadenopathy
8	Congenital anomalies of the pulmonary artery: a) Congenital proximal interruption of the pulmonary artery b) Peripheral pulmonary artery stenosis	a) Unilateral lung volume loss Mismatched perfusion defect on VQ scan b) Mismatched perfusion defects	a) Smooth abrupt cut off of the pulmonary artery within 1 cm of the hilum on CTA Unilateral perfusion defect on VQ scan Absence of findings of pulmonary hypertension b) Congenital anomaly Can be seen in Noonan's, Alagille's or William's syndromes	Presence of other cardiac anomalies

CTEPH: chronic thromboembolic pulmonary hypertension, VQ: ventilation perfusion, RVOT: right ventricular outflow tract, PET: positron emission tomography, CTA: computed tomographic angiography, MRI: magnetic resonance imaging, CXR: chest xray, PVOD: pulmonary veno-occlusive disease

### In situ thrombosis

In situ thrombosis of pulmonary arteries resembling chronic thromboembolism on CT scan can occur in patients with congenital heart disease with a left to right shunt (atrial septal defect, ventricular septal defect, patent ductus arteriosus and anomalous pulmonary venous drainage) and severe idiopathic PH.^9,10^ Anecdotally, we have also observed in situ thrombosis in patients with advanced pulmonary parenchymal or airway disease and secondary PH with dilated pulmonary arteries. In situ thrombosis is perhaps the most frequent CTEPH mimicker encountered by expert CTEPH centers.

The exact mechanism behind thrombus formation is poorly understood, but it has been hypothesized that multiple factors may play a role. These include shear stress on the vessel wall, chronic stasis in the aneurysmal pulmonary dilations, increase in blood viscosity, chronic hypoxemia and endothelial damage.^9,11,12^ In congenital heart diseases, due to progressive right-sided pressure and volume overload from the shunting, there is increase in shear stress which leads to endothelial dysfunction and progressive vascular remodeling; shunt reversal develops over time leading to Eisenmenger's physiology. There is a high risk (21 to 29%) of thrombus formation in this condition.^11,12^

**Fig. 1.**
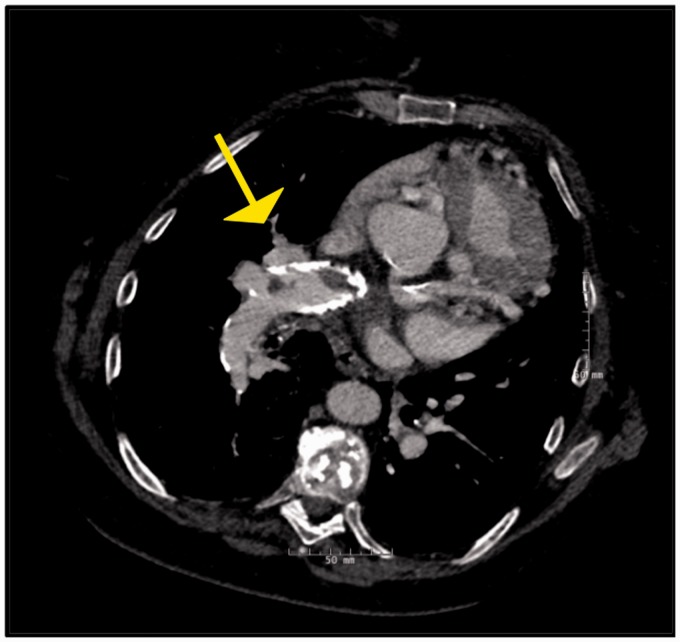
Eisenmenger's syndrome with eccentric mural pulmonary artery calcification.

**Fig. 2.**
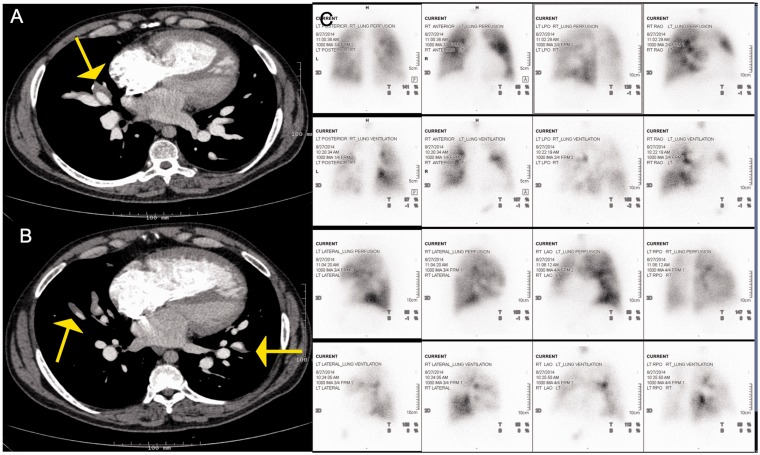
Panel A and B: Contrast enhanced CT images in a 50-year-old patient with in situ thrombosis in Eisenmenger's syndrome with an unrepaired atrial septal defect demonstrating multiple eccentric filling defects. Unlike typical webs of CTEPH, these are fairly smooth in appearance and the vessel appears normal in caliber. Panel C: Planar ventilation and perfusion images in multiple projections in the same patient. The perfusion images are more homogenous than the ventilation images in general. Multiple matched defects are noted in both lungs such as in the anterior segment of the RUL, RML, Superior segment of the RLL, Anterior segment of LUL, superior segment of the LLLL, and the posteromedial segment of LLL, No segmental mismatched defects are seen in either lungs to suggest CTEPH.

Clinically, it is difficult to diagnose in situ thrombosis as patients present with non-specific complaints. Chest radiography may show signs of PH like aneurysmal enlargement of the pulmonary arteries, pulmonary plethora and even pulmonary arterial calcifications (Fig. 1) in long standing cases.^12^ Echocardiography can detect intracardiac shunts, but shunts like sinus venosus defects, patent ductus arteriosus and anomalous venous return may be missed.^13^ A characteristic feature on CTA is neovascularization or the formation of small intrapulmonary vessels in the subpleural region near the centrilobular arterioles.^11^ This will manifest as diffuse ground glass opacities, patchy pulmonary opacities, and atelectasis.^14^ Thrombus can form in the central or distal pulmonary arteries and typically appears as a smooth lining thrombus attached to the artery wall in a vessel that is dilated (Fig. 2).^15^ This appearance of the thrombus can cause difficulty in distinguishing in situ thrombosis from CTEPH. Some features that can point to a diagnosis of CTEPH in these situations is the presence of mosaic attenuation in the lungs, peripheral parenchymal infarcts, and irregular vessel size.^16^ In situ thrombosis is non obstructive, which explains why VQ scans typically do not show perfusion defects (Fig. 2), a big clue to identify this as a non-CTEPH lesion.^15,17^ VQ scans will either be normal or show a mottled appearance in patients with PH with in situ thrombosis, whereas in CTEPH it will show segmental or lobar defects.^10^

The distinction between CTEPH and in situ thrombosis is critical, as pulmonary endarterectomy would not benefit these patients; it would not lead to any hemodynamic improvement, and will impose considerable surgical risk on these patients. Treatment of in situ thrombosis includes therapy with oxygen, PH-targeted therapies, and anticoagulation.^10,18^ Treatment of in situ thrombosis in patients with Eisenmenger's physiology is difficult because these patients have bleeding tendencies due to various reasons like clotting factor deficiencies, abnormal platelet function, and thrombocytopenia.^12^ Anticoagulation can provoke severe hemoptysis in these patients and some experts recommend against its use.^12^

### Pulmonary artery sarcoma

Pulmonary artery sarcoma (PAS) is a rare but aggressive malignancy of the pulmonary artery, which was first described by Mandelstamm in 1923.^19^ There are only a few hundred cases reported in the literature but this entity is now being increasingly recognized. PAS originates from the mesenchymal cells of the intimal layer of the pulmonary artery.^20^ Leiomyosarcoma, a subtype of sarcomas, originates from the medial layer of the pulmonary artery.^21^ The tumor usually occupies the entire lumen of the pulmonary trunk and progressively extends bilaterally towards the main branches of the pulmonary artery, sometimes causing extraluminal tumor expansion.^20^ It is thought to arise from the pulmonary trunk but is adherent to multiple sites and can be found to involve the pulmonary valve as well as the right ventricular outflow tract.^21^

Due to its slow growth, the symptoms are subacute to chronic in nature.^20^ The symptoms of sarcoma are non-specific and include dyspnea, chest pain, cough, hemoptysis, fatigue, weight loss, dizziness and syncope.^19,22^ Patients often present at an advanced stage of their disease with signs of PH and right heart failure.^23^ In the laboratory analysis, patients with PAS were found to have lower levels of D-Dimer and BNP than in patients with chronic thromboembolic disease.^24^

**Fig. 3.**
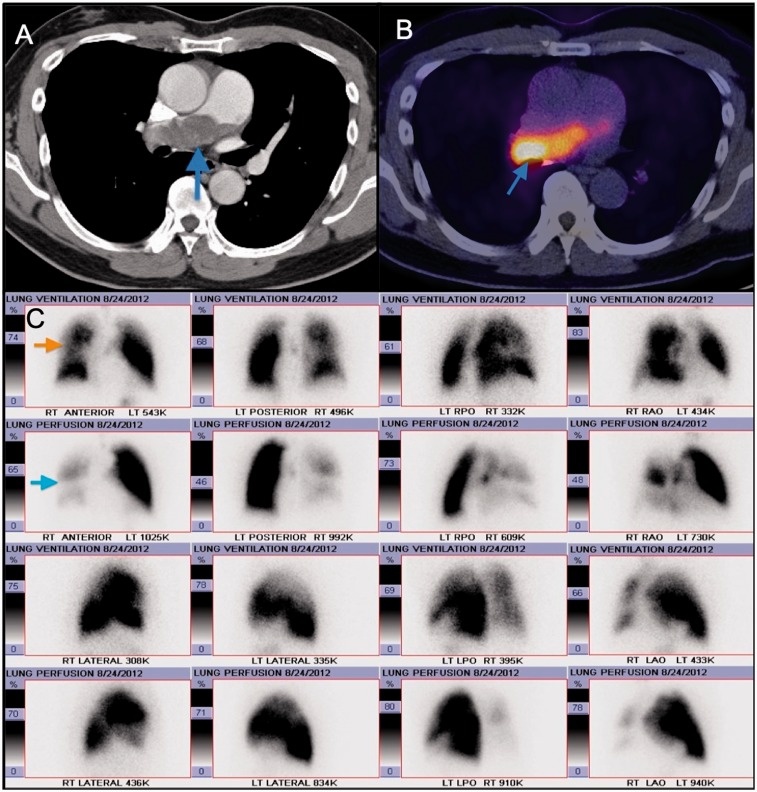
Panel A and B: Axial contrast enhanced CT mage showing a large filling defect with a lobulated appearance in the main pulmonary artery extending to the right main PA. Corresponding fused PET-CT image showing FDG uptake in the lobulated mass in the right main pulmonary artery, consistent with neoplastic process. Panel C: Images from corresponding planar ventilation-perfusion scan demonstrate mismatched perfusion defects in the right lung with severely compromised perfusion.

The presence of multiple mismatched perfusion defects on a VQ scan (Fig. 3) and pulmonary arterial filling defects on a CTPA can mimic CTEPH. Due to similarities in the clinical features and imaging findings of PAS and CTEPH, the two conditions are often difficult to distinguish; and PAS is frequently misdiagnosed leading to delays in diagnosis and treatment. According to a review of 391 patients with PAS, 45 (11%) patients were misdiagnosed as CTEPH before being diagnosed as having PAS.^19^

On computed tomography, PAS presents as a heterogeneously enhancing low-attenuation filling defect frequently occupying the entire diameter of the pulmonary artery trunk or the main pulmonary arterial branches (Fig. 3) causing expansion of the involved arteries, invasion of the tumor into the pulmonary arterial wall, and extravascular tumor extension.^19,22^ While these findings are non-specific and can be seen in pulmonary thromboembolism with a large clot burden,^20^ they are not typically seen in CTEPH and should prompt the suspicion of PAS. The tumor can also appear as multiple endoluminal filling defects in the pulmonary arterial branches, which can lead to delays in diagnosis and treatment as this appearance can be confused with that of acute or chronic thromboembolism.^22^ Kim et al.^24^ studied the CT findings of patients with PAS and pulmonary thromboembolism, and found that patients with PAS demonstrate larger size of mass along with heterogeneous appearance, tumoral impaction, lobulated or bulging contour, cauliflower-like polypoidal appearance, wall eclipse sign (PAS arises from the wall of the artery eclipsing it at least on one side), intra-tumoral vessels, central location and lung ischemia. These findings were found to be significantly more frequent in PAS versus pulmonary embolism.^24^ Based on a comparative study of 7 pulmonary sarcoma patients and 40 patients with pulmonary thromboembolism, PASs are more likely to be unilateral compared to thromboembolic disease; and are also commonly heterogeneous in appearance due to factors like tumor necrosis, hemorrhage, and calcification.^21,22^ PAS appears as a delayed contrast enhancement on CT angiography scans, more evident in the venous phase.^21,24,25^ The presence of multiple lung nodules can raise a suspicion for a malignancy like PAS.^19^

The diagnosis of PAS should be suspected in patients with insidious symptoms, absence of a history of deep vein thrombosis or pulmonary embolism, large unilateral filling defect in the proximal pulmonary artery, heterogeneous appearance, enlarging filling defect after effective anticoagulation, and presence of pulmonary nodules.^20,22^ One of the important distinguishing features of PASs is their PET avidity and this test should be ordered if any of the above features are present.^20,26^ PAS demonstrates increased FDG (18 F-fluorodeoxyglucose) uptake on positron emission tomography (PET) scans, whereas CTEPH does not show PET avidity.^23^ A SUV cut-off of 3.5 has been shown to have a sensitivity, specificity, and accuracy of 100% for the diagnosis of PAS.^8^ While the above is usually true, it is important to be aware of certain exceptions. There are two case reports described in the literature wherein PAS demonstrated low FDG uptake; this has been hypothesized to be as a result of surrounding thrombus, cystic changes in leiomyosarcoma or thin walls of pulmonary aneurysms.^21,24^ Pulmonary infarctions in CTEPH can demonstrate increased FDG uptake and can be mistaken for metastatic nodules.^8^ Nevertheless, FDG-PET scan remains an important modality to differentiate PAS from CTEPH.

MRI can be a helpful problem-solving tool in the evaluation of PAS. Diffusion-weighted imaging (DWI) demonstrates areas of restricted diffusion in PAS due to increased cellularity, a finding not seen with bland thrombus. Also, post-gadolinium MRA can demonstrate delayed heterogeneous enhancement of the PAS, which is not seen in avascular lesions like thromboembolism.^24^

Transthoracic echocardiography may show the presence of pulmonary regurgitation secondary to involvement of the leaflets by the tumor. A transesophageal echocardiogram is also a valuable tool in identifying tumor invasion into the arterial wall.^19^ Occasionally, PAS can be seen moving with the cardiac cycle on an echocardiogram if it is attached to the wall at one place.^27^ This feature can differentiate a PAS from pulmonary embolism since thrombi do not move with the cardiac cycle.

Confirmation of diagnosis requires a surgical biopsy or a frozen section, but more often than not patients require a diagnostic and therapeutic surgery. There has been a report of endobronchial ultrasound (EBUS)-guided transbronchial needle aspiration (TBNA) through the pulmonary artery helping in making a diagnosis.^28^ The prognosis of PAS is extremely poor and death can occur within weeks to months of diagnosis due to right heart failure secondary to right ventricular outflow tract obstruction or distal embolization.^19,25^ Mean survival has been reported to be between 12 and 18 months although there are certain cases reported in the literature with survival of up to 102 months.^21,23,25^ Survival in those who do not undergo surgery has been reported to be <2 months.^20^ Aggressive surgical resection appears to be the only effective modality of treatment at present and has been reported to increase survival.^25^ Reported surgical treatments include lobectomy or pneumonectomy for unilateral cases, pulmonary endarterectomy, and tumor debulking with or without graft reconstruction of the pulmonary artery for bilateral cases.^23,25,29^ The goal of pulmonary endarterectomy is to relieve the obstruction caused by the tumor, which causes significant improvement in symptoms but this is not curative.^23^ The role of chemotherapy and radiation post-surgery is yet unclear.^23^ Even in patients that have undergone heart-lung transplantation, distant metastases have occurred leading to poor outcomes.

### Pulmonary vein involvement

#### Fibrosing mediastinitis

Fibrosing mediastinitis (FM), also known as sclerosing mediastinitis is a rare but potentially lethal disease characterized by proliferation of fibrous tissue in the mediastinum and around the hila.^30^ It can cause progressive fibrosis with compression and occlusion of mediastinal structures like the airways, esophagus, pulmonary vasculature, superior vena cava and phrenic and recurrent nerves.^30,31^ It is thought to be caused by an exaggerated host response to prior infection with granulomatous organisms. A vast majority of cases are secondary to infection with the fungus *Histoplasma capsulatum*; other causes include tuberculosis, aspergillosis, cryptococcosis, mucormycosis, blastomycosis and even non-infectious conditions like sarcoidosis.^30–32^ Other causes include mediastinal radiation, systemic lupus erythematosus, rheumatoid arthritis, Behcet's disease, and a rare idiopathic form without known triggers.^30,32^ It can be associated with significant morbidity depending on the extent of fibrosis.^30^

Clinical features include young age at presentation, dyspnea, cough, sputum production, pleuritic chest pain, history of recurrent pulmonary infections, pulmonary edema and hemoptysis.^30,33^ Symptoms vary depending on the structures affected by fibrosis.

**Fig. 4.**
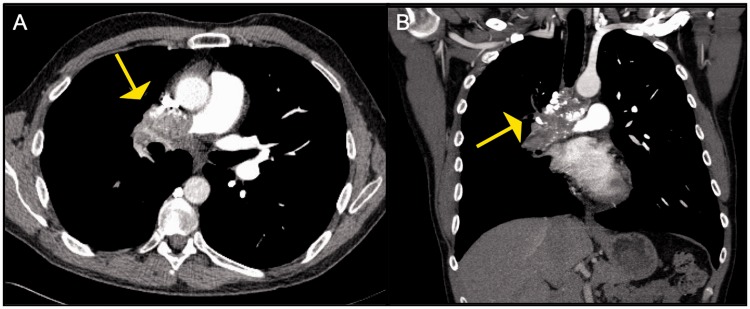
Pulmonary artery invasion seen in a patient with fibrosing mediastinits: Axial CT image demonstrates a heterogenous partially calcified mass invading the right PA. This mass was biopsied and was found to be granulomatous in nature.

FM can cause severe PH due to extrinsic compression of pulmonary vasculature (Fig. 4) and can closely resemble CTEPH radiologically.^30^ Both conditions can demonstrate large mismatched perfusion defects on a VQ scan but they can be distinguished from each other based on other imaging modalities.^5^ On chest radiographs, FM can present as mediastinal widening with or without lymphadenopathy.^30,33,34^ Features of FM on chest CT include a diffuse infiltrative soft tissue process in the mediastinum that may also extend to the bilateral hila and the pulmonary parenchyma.^30^ The middle mediastinum, particularly the subcarinal and the paratracheal region is most commonly affected.^33^ The fibrotic tissue has variable contrast enhancement and is frequently calcified.^31,33,34^ It can also present as a focal mediastinal mass with stippled calcification in the paratracheal or subcarinal region instead of a diffuse process.^30,33,34^ Indeed, extensive mediastinal calcification is a good clue to the diagnosis of FM but, the main distinguishing features of FM from CTEPH is the compression of multiple structures other than the pulmonary arteries.^29^ Bronchial stenosis and bronchial wall thickening can result from extrinsic compression of the airways by fibrosis. This can cause lobar or segmental atelectasis and/or post obstructive pneumonia.^30,31^ Pulmonary venous compression is another complication of FM that can cause interstitial septal thickening, Kerley B lines, pulmonary edema, and pleural effusions.^30–32^ Extrinsic compression of the pulmonary vasculature can cause pulmonary infarcts, which look like wedge-shaped pleural-based opacities.^33^ Longstanding obstruction of the pulmonary arteries or veins can cause PH and cor pulmonale and mimic CTEPH.^31^ In a study of 27 cases of fibrosing mediastinits with associated PH, severe extrinsic compression of the pulmonary arteries was seen in 22 patients and severe pulmonary venous compression was seen in 14 cases.^35^ FM is also reportedly the most common benign cause of superior vena cava obstruction in the United States.^30^

One of the ways to differentiate FM from CTEPH (besides the mixed bronchovascular involvement) is that the arteries tend to be narrowed and stenosed centrally, specifically in areas of soft tissue infiltration. The more distal arterial vasculature is smooth and does not demonstrate the undulation and areas of stenoses that can be seen with CTEPH.^36^ Bronchoscopy is usually needed for therapeutic purposes in FM for management of airway complications. Features on bronchoscopy include widening of the carinal bifurcation, bronchial stenosis, or concentric narrowing. In patients with histoplasmosis, mucosal edema, hyperemia, and anthracotic pigmentation have been described.^30,37^

Treatment is challenging and very few therapeutic options are available. Anti fungal therapy or anti tuberculous therapy may be attempted in cases with histoplasmosis or tuberculosis respectively but there is no robust data proving the efficacy of these treatments.^30^ Percutaneous balloon angioplasty or stenting can be tried for stenosis of the pulmonary vasculature or the SVC but these procedures are associated with a high morbidity and mortality.^31,38^

## Pulmonary vein stenosis secondary to ablation for atrial fibrillation

Pulmonary vein stenosis (PVS) is a rare condition that is becoming more common with the advent of radiofrequency ablation as a treatment for atrial fibrillation.^39,40^ Other causes include congenital heart disease and mediastinal tumors.^39,40^ It is characterized by progressive luminal narrowing of the pulmonary veins.^40^ PVS can occur days to months following the ablation procedure but the mean has been reported to be somewhere between two and seven months.^41^ Clinically, it can present as shortness of breath, cough, chest pain or hemoptysis in patients with more severe (>50%) stenosis.^40,42^ A prior history of ablation should prompt a consideration of pulmonary venous stenosis as a cause of symptoms.

**Fig. 5.**
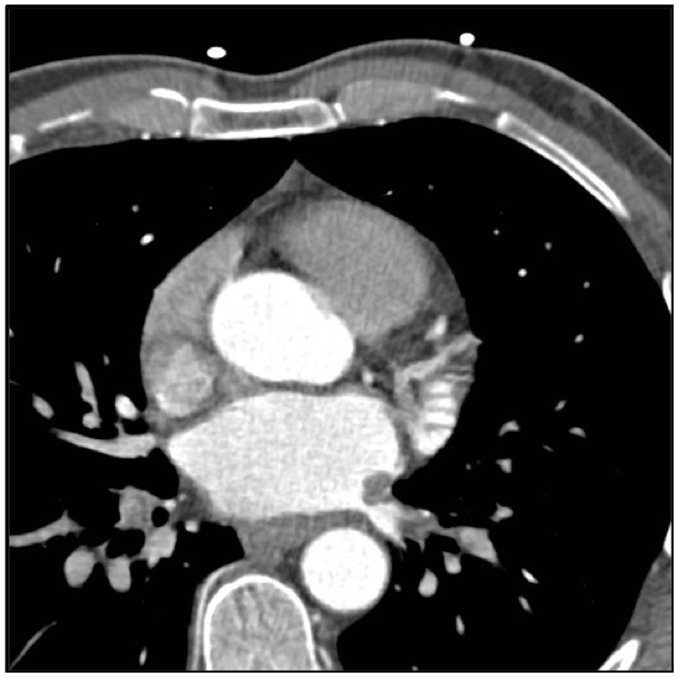
Pulmonary vein stenosis secondary to ablation for atrial fibrillation: Axial CT image from a CT-pulmonary vein protocol study demonstrates at least moderate stenosis of the left inferior pulmonary vein close to its ostium.

Up to 80% of patients with pulmonary venous stenosis can present with multiple mismatched perfusion defects on a VQ scan due to obstruction to blood flow and mimic pulmonary thromboembolism.^40,43^ Due to increased pressures in the pulmonary veins, there is longer blood/contrast transit time in the lung that is drained by the stenosed vein causing decreased perfusion to that part of the lung.^44^ It can also cause non-opacification of segmental and subsegmental veins of the lung (Fig. 5), which, if mistaken for the pulmonary arteries, can mimic pulmonary thromboembolic disease.^44^ PVS can present with findings of venous congestion on chest radiography, and demonstrate the presence of Kerley B lines, diffuse interlobular septal thickening, and pleural effusions.^43^ These findings are not present in CTEPH. CT-PV protocol with enhancement timed to left atrium is currently the imaging modality of choice for diagnosis of PVS due to its high spatial resolution and multiplanar views that can be obtained in a short scanning time.^39,40^ It can be used to distinguish between PVS and CTEPH. MRI can also be used to reliably detect the venous stenotic lesions but is limited by the longer scanning time and lower spatial resolution compared to a CTA.^39,40,44^ Transthoracic echocardiography can be used for diagnosing PVS but is not as sensitive as a CTA in adults.^44^

Management of PVS includes balloon angioplasty with or without stenting in symptomatic cases with severe stenosis.^40,45,46^ There are no definite guidelines on the management of asymptomatic patients with severe stenosis; however, a study by Di Biase suggested that an early intervention in these patients is more likely to prevent progression to permanent total venous occlusion and development of severe PH.^46^ Prognosis of PVS is not favorable and restenosis rates of 25% and 49% have been reported after stenting and balloon angioplasty respectively.^42^

## Pulmonary veno-occlusive disease/pulmonary capillary hemangiomatosis

Pulmonary veno-occlusive disease and pulmonary capillary hemangiomatosis are rare diseases that occur due to obstruction of the pulmonary veins, venules, or capillaries.^47^ They are increasingly recognized causes of PH. Both conditions affect young adults with a slight male preponderance for PVOD.^47,48^ Clinical presentation mimics most other causes of PH and includes gradually progressive dyspnea, exercise intolerance, and leg edema. Right heart catheterization characteristically shows increase in the pulmonary arterial pressures with a normal or a low wedge pressure.^49^ Use of a pulmonary vasodilator may lead to acute pulmonary edema due to an increase in the transcapillary pressure gradient secondary to pulmonary arterial vasodilation without concomitant pulmonary venodilation.^47,49^

The VQ scan in both these conditions is often normal but there have been reports of a mottled pattern mimicking pulmonary arterial hypertension, multiple mismatched segmental perfusion defects mimicking CTEPH as well as a unilateral large perfusion defect due to asymmetric involvement of the pulmonary veins.^48,50^ For example, Bailey et al.^48^ reported a case series of three patients with high probability VQ scans in patients with PVOD. Seferian et al.^50^ reported mismatched perfusion defects in 4 out of 56 (7.1%) patients with PVOD. CT findings of PVOD/PCH are fairly useful in differentiating it from CTEPH, and common features include widespread smooth interlobular septal thickening, ground glass centrilobular opacities, and mediastinal adenopathy.^47,51^ Pleural effusions and general signs of PH can also be present.^48^ Pulmonary angiography usually shows dilated pulmonary arteries, normal left atrium, and normal pulmonary veins.^48^ Compared to other forms of PH, PVOD/PCH presents with a lower DLCO and more severe hypoxemia.^51^

A highly probable diagnosis of PVDO/PCH can be made with right heart catheterization and the classic imaging findings on CT scan along with the typical sings of decreased DLCO and hypoxemia. A definitive diagnosis requires a biopsy, which often is too risky to perform.^49^ Diagnosis is often made post mortem. The only cure for this condition is lung transplantation. PH-targeted therapies, anticoagulation and diuretics can be used for palliative purposes.^49^

## Large vessel vasculitis

Large vessel vasculitis involving the pulmonary arteries can be seen in Takayasu's arteritis, giant cell arteritis, and Behcet's disease. Pulmonary arterial vasculitis can mimic CTEPH as it can cause extrinsic compression as well as endoluminal obstruction of the pulmonary artery.^52,53^

Takayasu's arteritis (TA), also know as the ‘pulseless disease’, is a rare chronic vasculitis of unknown etiology that mainly affects the aorta and its branches but it can also involve the pulmonary arteries.^54,55^ Although symptomatic pulmonary involvement is relatively uncommon in TA, studies have shown involvement of the large and medium pulmonary arteries in roughly 50% of patients with this condition.^54^ Isolated involvement of the pulmonary arteries is rare.^56^ It is predominantly seen in young women under the age of 40 years of East Asian ancestry.^56,57^

Clinically, presentation is nonspecific, often insidious and depends on the site, extent, and duration of vascular involvement.^56,58^ Patients can present with generalized symptoms of fever, night sweats, malaise, weight loss, arthralgias, and myalgias.^56^ They can also present with signs of organ ischemia like myocardial ischemia or infarction, stroke, uncontrolled hypertension, and visual disturbances due to involvement of the myocardial, cranial, renal, or retinal vessels respectively.^56,59^ Limb claudication, diminished, or absent pulses and vascular bruits may also be present due to peripheral arteriopathy.^56,59^ Pulmonary bruits over the lung fields have been described in patients with CTEPH but these patients do not have bruits over other systemic arteries.^59^ Patients may also present with respiratory symptoms like shortness of breath, cough, chest pain, and hemoptysis.^56,57,60^ The development of PH can occur in TA but its incidence is about 12-13%.^58^ Variable disease progression and nonspecific signs and symptoms often lead to a delayed diagnosis.^56^ Increase in laboratory markers of inflammation like erythrocyte sedimentation rate and C- reactive protein can be seen although a negative value does not rule out vasculitis.^56^ Anemia is also often seen in patients with TA where in CTEPH, the hematocrit is often normal to elevated.^59^

**Fig. 6.**
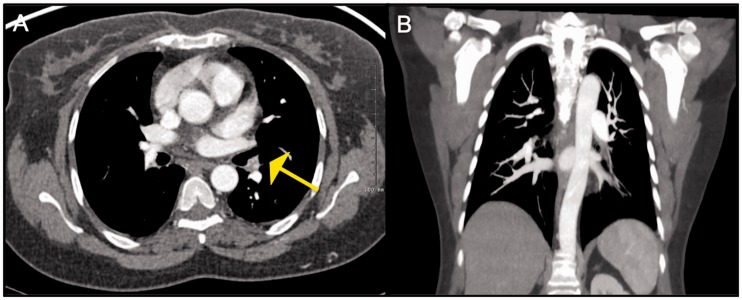
Patient with Takayasu arteritis with CT image showing severe stenosis of the lingular segmental branch.

Diagnosis is based on clinical presentation and various imaging modalities.^56^ Computed tomographic angiography and MRI are considered the tests of choice for TA as they help assess not only the arterial lumen but also the arterial wall changes.^61^ On CTA, pulmonary vasculitis typically presents with concentric circumferential arterial mural thickening with high attenuation on pre contrast and double ring enhancement pattern on post contrast imaging.^56,62^ Other features include transmural calcium deposition, luminal stenosis (Fig. 6) and/or dilation and less commonly occlusion, ectasias, and aneurysms of involved arteries.^56,62^ The pulmonary arterial pseudoaneurysms may be associated with in situ thrombus formation that may mimic CTEPH.

On pulmonary angiography, different findings like stenosis, dilation, occlusion and luminal irregularities can be seen in large vessel vasculitis as well as CTEPH.^59^ A study of 48 patients with TA and PH showed that the main pulmonary artery was more often involved compared to the distal pulmonary arteries in TA, whereas another study of 98 patients showed more frequent involvement of the segmental and subsegmental branches.^54,60^ Right-sided involvement and involvement of the upper lobe branches of the pulmonary arteries are also more common than left sided or lower branch involvement.^54,60^ A thin, beaded appearance of the pulmonary arteries can suggest TA over CTEPH. Although angiography is a useful evaluation tool, assessment of the vessel wall with this modality is suboptimal.^61^ Angioscopy can help differentiate between TA and CTEPH, though it is rarely done. In CTEPH, the intima looks irregular and pitted while in arteritis, its appearance is smooth and uniform.^59^ Formation of systemic to pulmonary shunts distal to occluded pulmonary arteries has also been described in TA and their formation suggest extensive involvement of the pulmonary arteries.^54,56^ This feature though, is also seen in CTEPH and is not helpful in differentiation.^59^ A PET scan also plays a valuable role in diagnosis of TA as pulmonary vasculitic lesions will demonstrate increase in FDG uptake compared to non-inflamed vessels.^61,63^

Imaging of the aorta and its branches, via MRI or conventional angiography, is critical to the diagnosis of TA. On aortography, aortic stenosis, dilation, aneurysms, and occlusion may be seen.^42,54^ Some vessels may have a beaded appearance and others may show abrupt tapering with pre-stenotic dilation.^59^

The main distinguishing features of TA from CTEPH are the presence of systemic symptoms and signs, particularly arterial symptoms such as limb claudication, neurologic deficits, and diminished systemic pulses, and imaging evidence of aortic involvement. Both aortography and pulmonary arteriography are necessary to establish a confident diagnosis and evaluate the extent of the disease.

Patients who develop PH secondary to pulmonary arteritis in TA have a very poor prognosis.^56^ The mainstay of treatment for TA is high dose glucocorticoids.^57^ Methotrexate, azathioprine and in severe cases, cyclophosphamide and infliximab have been used.^56^ Percutaneous transluminal angioplasty, stenting or bypass surgery may also be considered for critical vessel stenosis.^56,62^

## Sarcoidosis

Sarcoidosis is an idiopathic chronic inflammatory condition that can affect multiple organs but the most commonly involved organ is the lung.^64^ PH is highly prevalent in sarcoidosis and can be present in up to 28% of cases.^65^ Sarcoidosis can cause PH through multiple mechanisms, the most common cause being pulmonary parenchymal fibrosis. Other mechanisms include direct involvement of the vessel wall with granulomas, granulomatous FM (as described in detail above), and extrinsic compression of pulmonary vessels from mediastinal lymphadenopathy.^66^ A small case series by Tandon et al.^67^ raised the hypothesis that sarcoidosis may also be associated with CTEPH due to the increased risk of venous thromboembolism, and may be another mechanism of developing PH in these patients.

Sarcoidosis is often termed ‘the great mimicker’ as the clinical presentation and imaging findings may be non-specific or atypical. A VQ scan may show mismatched perfusion defects suggesting CTEPH.^39^ Also, certain features seen on CTA in CTEPH like arterial webbing, intimal irregularities, and abrupt vascular narrowing with post stenotic dilation may also be seen in sarcoidosis-associated PH due to granulomatous involvement of the vessels.^67^ Hence, one must be aware of other characteristic features that can help differentiate the two disease conditions.^67^

The appearance of pulmonary sarcoidosis on imaging depends on the stage of presentation. Stage I disease presents with mediastinal lymphadenopathy commonly involving the hilar and paratracheal nodes.^64^ The lymphadenopathy is usually bilateral symmetrical and non-necrotic.^64^ Other locations for nodal involvement include sub aortic, AP window nodes, and subcarinal nodes. In long standing disease, nodal calcification is commonly seen. Stage II disease involves the pulmonary parenchyma along with mediastinal lymphadenopathy. Stage III involves the lung parenchyma without any lymphadenopathy. The hallmark of pulmonary sarcoidosis is the presence of lung nodules in a peribronchovascular and perilymphatic (subpleural area and along the interstitium) distribution. The upper lobes of the lungs are the most frequently involved.^64^ Sometimes the nodules can coalesce and form larger nodules or less commonly, large mass-like areas. Stage IV disease is end stage fibrotic lung disease with fibrosis, traction bronchiectasis, and subpleural honeycombing predominantly involving the bilateral upper and mid lung zones. Although PH is more commonly associated with advanced stages of sarcoidosis, it can be seen in patients with Stage 1 disease as well.^68^ Alveolar sarcoidosis is a rarer form that can present with consolidative opacities on the Chest CT.^64^ Another uncommon form of sarcoidosis called miliary sarcoidosis presents with diffuse multiple tiny miliary nodules. Mediastinal lymphadenopathy and parenchymal involvement are helpful features in differentiating sarcoidosis from CTEPH.

**Fig. 7.**
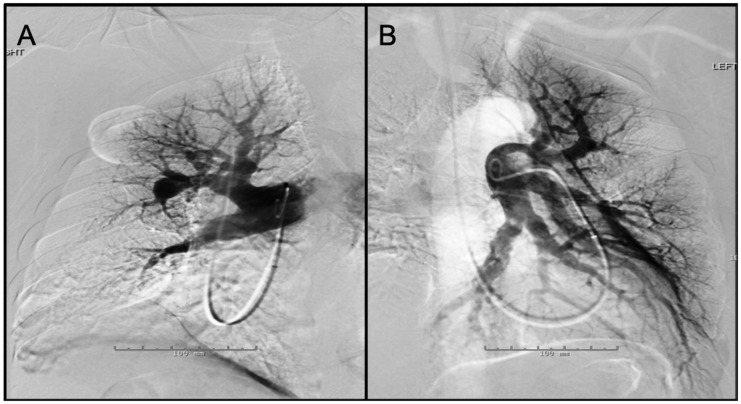
Panel A: Digital subtraction image from a right pulmonary angiogram in LAO 42 ° projection – There is stenosis of anterior segmental branch of RUL with post-stenotic dilation. Also noted is atrophic basilar segmental branch of the RLL. The perfusion in the periphery of the RUL (anterior segment) is diminished. Panel B: Digital subtraction image from a left pulmonary angiogram in RAO 37 ° Caudal 1 projection – There are atrophic/occluded lingular segmental branches. The basilar segmental branch of the LLL demonstrates luminal irregularities without significant luminal narrowing. There is associated decreased perfusion of the lingula.

**Fig. 8.**
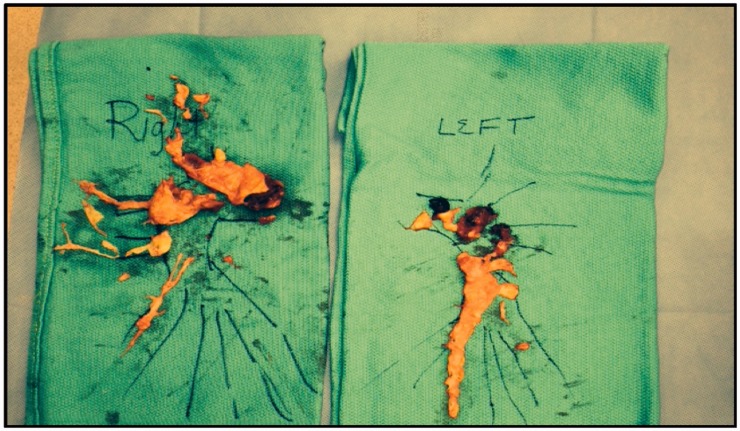
Surgical specimens of the right and left pulmonary arterial chronic clots, with the largest burden in the RUL.

**Fig. 9.**
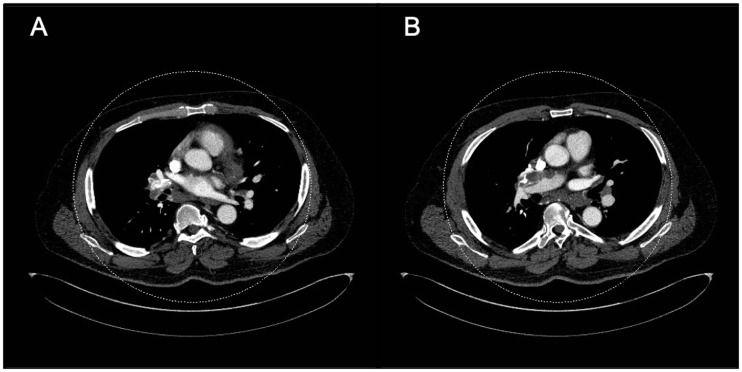
Axial CT images in the mediastinal window settings demonstrate calcified confluent mediastinal and right hilar lymphadenopathy in a patient with combined CTEPH and sarcoidosis. Also noted is lobular filling defects in the right main and interlobular pulmonary artery, consistent with CTED; there is mass effect on the right interlobar PA secondary to the calcified lymphadenopathy.

**Fig. 10.**
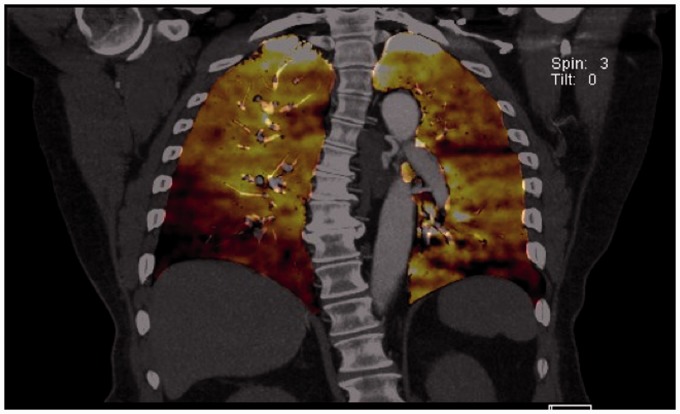
Corresponding perfused blood volume (PBV) image coronal projection in the patient with CTEPH and sarcoidosis demonstrates peripheral wedge-shaped perfusion defects in both lower lobes, right greater than left.

Of note, as mentioned above, sarcoidosis and CTEPH can co-exist in the same patient (Fig. 7 to 10). It is crucial to rule out CTEPH in any patient with sarcoidosis-associated PH because, if present, PEA can be used to treat these patients.^67^ CTA will show absence of filling defects in the pulmonary arteries if PH is caused due to extrinsic compression of the vessel as opposed to CTEPH.

Sarcoidosis, in general, carries a favorable prognosis but when associated with PH, prognosis is poor and carries a significant morbidity and mortality.^68^ Treatment of sarcoidosis associated PH is not well established and includes anti-inflammatory therapies, pulmonary vasodilators, anticoagulation, and oxygen.^68,69^

## Malignancy

**Fig. 11.**
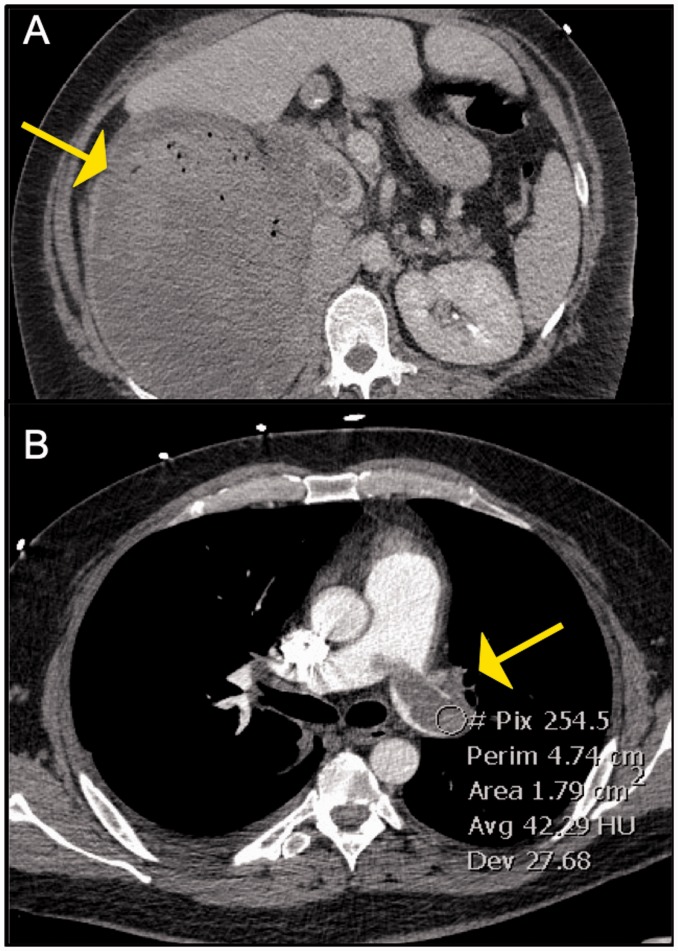
Panel A: Axial image from CT abdomen showing a large heterogenous right renal mass which was found to be renal cell carcinoma Panel B: Corresponding image from CT-PE protocol exam showing an enhancing filling defect in the left main PA. This was surgically removed and found to be a tumor thrombus.

Pulmonary tumor thrombotic microangiopathy (PTTM) is a condition in which tumor cells embolize to the pulmonary circulation leading to activation of the coagulation cascade, thrombus formation, intimal proliferation, obstruction and subsequently PH.^70^ Tumors commonly associated with tumor embolization include renal cell cancer (Fig. 11), soft tissue sarcomas, and atrial myxomas, but it has also been reported in multiple other tumor types like breast cancer, gastric cancer, liver cancer, thyroid cancer, testicular cancer, and uterine cancer.^9^ In an autopsy study by Winterbauer et al.,^71^ tumor embolization was found in 26% of patients with cancer and in about 8% of the cases, it was a significant cause of death of the patient.^71^ We reported a case of right atrial myxoma metastases to the pulmonary arteries giving the appearance of chronic thromboembolic lesions.^72^ PTTM can also occur due to direct tumor extension to the pulmonary arteries through the right heart chamber or the vena cavae.^70^ A frequent cause of complete lack of perfusion of one lung on VQ scan is lung cancer.^73^

Patients can present insidiously with symptoms of dyspnea on exertion, cough, chest pain, hemoptysis as well as general symptoms like fatigue, malaise, night sweats, and weight loss. Most of these patients are also hypoxic.^70,74^ Although cough may be present in CTEPH, it is not a common symptom like in PTTM.^70^ In more advanced cases, patients can present with decompensated heart failure.^70^ On laboratory analysis, signs of microangiopathic hemolytic anemia like thrombocytopenia, anemia, elevated D-dimer, and elevated LDH may be seen.^70^

The VQ scans of these patients typically show multiple symmetric peripheral subsegmental mismatched perfusion defects that are distributed evenly throughout the lungs.^75,76^ This VQ scan pattern can also be seen in idiopathic PH, septic emboli, fat emboli, and talc granulomatosis in IV drug abusers.^75^ This is different from the larger mismatched perfusion defects seen in the larger pulmonary arteries in CTEPH although features overlap and diagnosis is not conclusive.^77^ In PTTM, there are no specific diagnostic signs on a Chest CT but there may be ancillary findings related to the tumor that may prove helpful in pinpointing the diagnosis.^76^ A Chest CT scan may show asymmetric intra and interlobular septal thickening that may be nodular in appearance secondary to lymphangitic infiltration by tumor cells or tumoral edema.^70,76^ There may also be mediastinal or hilar lymphadenopathy or lung nodules, which may represent metastatic disease. A case series reported four patients with a ‘dilated and beaded’ appearance of the peripheral pulmonary arteries due to tumor emboli.^76^ Other features include ground glass opacities and centrilobular tree in bud opacities.^70^ Features of PH including right-sided heart enlargement and enlargement of the pulmonary artery may be seen.^70^ PET avidity has been reported in some cases of PTTM depending on the lesion size and histological type of cancer. Thrombus in CTEPH is not PET avid and this may be a useful test to distinguish between the two entities if positive in PTTM.^74^ Pulmonary angiography is not an ideal modality for the evaluation of tumor emboli and has a very low sensitivity and specificity for diagnosis.^76^ A definite diagnosis may be difficult and requires tissue sampling and cytological analysis.^74^ A prior history of malignancy or current malignancy should prompt physicians to consider this diagnosis and investigate it with further work up. Finally, it is important to note that malignancy too, like sarcoidosis independently increases the risk of venous thromboembolism and can promote the development of CTEPH.^78,79^

The prognosis of patients with PTTM is extremely poor with death occurring within a few days to weeks due to right heart failure.^70^ If diagnosed early, treatment should be directed at the underlying malignancy. Pulmonary vasodilators, anti inflammatory agents like steroids and anti-proliferative agents targeting growth factors have been used but evidence is limited and is based on case reports.^78^

## Congenital anomalies of the pulmonary arteries

### Unilateral proximal interruption of the pulmonary artery

Unilateral proximal interruption of the pulmonary artery (UPPA) is characterized by abrupt termination of the pulmonary artery at the level of the hilum.^80^ This is a rare congenital anomaly with an incidence of 1 in 200,000 persons.^80,81^ The right side is more commonly affected than the left side. Left-sided interruption of the pulmonary artery is often associated with concurrent congenital cardiac anomalies like tetralogy of Fallot, right aortic arch, aortic coarctation, patent ductus arteriosus, septal defects, and transposition of great arteries.^81–83^ Right-sided defects are usually present in isolation.^18^ The affected lung is supplied by systemic collateral vessels formed mainly by branches of the bronchial arteries but also by collaterals from the intercostal, internal mammary, subclavian, subdiaphragmatic, or innominate arteries.^80,81,83^

This condition is typically diagnosed at a younger age (median age of 14 years).^83^ The most common symptoms include recurrent infections, dyspnea, exercise intolerance, and hemoptysis.^80,83^ In a significant proportion of adult patients, this condition can be asymptomatic.^84,85^ Rupture of the collateral vessels may occur as a complication in about 10 to 20% of patients causing pulmonary hemorrhage which can vary from self-limited to massive life-threatening hemorrhage.^80,81,83^ On physical exam, the presence of flow murmurs has been described limited to the affected side secondary to turbulent vessel flow. Flow murmurs over the lung fields can also be heard in patients with CTEPH.^84^ The incidence of PH in this condition is variable and reportedly varies from 19 to 44%.^81,83^

**Fig. 12.**
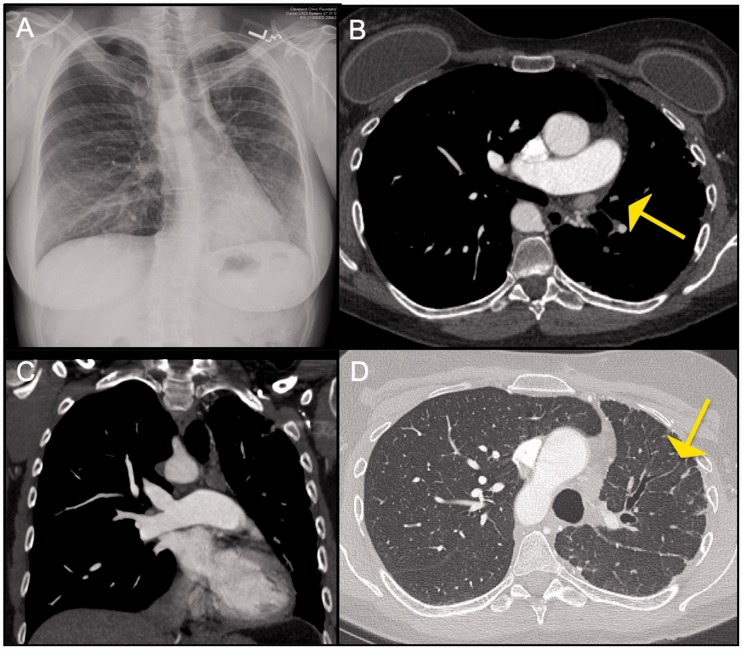
Panel A: CXR showing mediastinal shift to the left with volume loss in a patient with congenital interruption of the left pulmonary artery. Also noted is shallow appearance of the left hilum. Panel B and C: Axial CT images in mediastinal window settings demonstrate complete absence of the left PA. Few bronchial artery collaterals are noted in the left hilar region. Panel D: Corresponding lung window image shows volume loss of the left lung with few peripheral reticulations (which reflect nonspecific fibrosis).

**Fig. 13.**
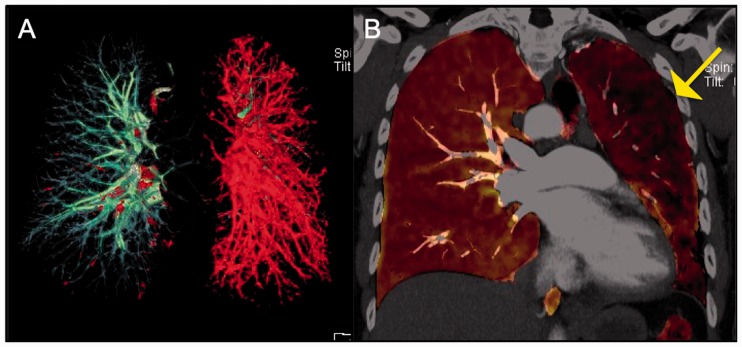
Perfused blood volume images from Dual energy CT data demonstrate near complete absence of the perfusion to the left lung.

UPPA is often suspected after a chest radiograph is obtained for other purposes and shows abnormalities suspicious of this condition. On a plain chest radiograph, the mediastinum is shifted to the affected side, the ipsilateral hemidiaphragm may be elevated and there is compensatory hyperinflation of the lungs on the unaffected side.^80,83,84^ The hilar shadow is absent.^86^ Other signs may be seen due to the collateralization of vessels on the affected side such as subpleural reticular opacities, rib notching due to intercostal arteries, and pleural thickening or parenchymal bands due to transpleural collaterals.^80,83^ However, not all patients present with these classic chest X-ray findings. Sometimes, normal or increased vascularity of the affected lung may be seen due to compensatory increase in bronchial collaterals or back perfusion of pulmonary arteries by these collaterals.^84^ Chest radiography in CTEPH may show similar findings of hypovascularity and unilateral lung volume loss due to prior pulmonary infarctions and can be confused with UPPA. A CT angiography is the preferred diagnostic modality as it can delineate the vascular anatomy more accurately.^80,83^ Abrupt termination of the pulmonary artery is seen at or within 1 cm of the hilum (Figs. 12 and 13). The CTA will also show the collateral vessels on the affected side of the lung and ipsilateral volume loss.^83^ If a main pulmonary artery is blocked by a thrombus in CTEPH, it may mimic UPPA on a CTA and be difficult to distinguish from this condition. A VQ scan may not be reliable in distinguishing UPPA and CTEPH as both conditions will show mismatched perfusion defects.^87^ However, in UPPA, the perfusion defect will only be seen on one side.^83^ Cardiac MRI may be useful in identifying other congenital anomalies of the cardiac structures that may be present concomitantly.^80^ A history of deep vein thrombosis may provide a clue to the presence of CTEPH. On physical examination, patients with CTEPH may demonstrate signs of PH and right ventricular failure which is not seen in patients with UPPA.^84^

Traditionally, a pneumonectomy is considered to be the defnitive treatment although it carries a high risk of morbidity and mortality.^81^ Embolization of collateral arteries in cases of hemoptysis has shown to be successful in 73–99% of patients according to a study with recurrence also relatively common occurring in up to half of the patients.^88^

### Peripheral pulmonary artery stenosis

Peripheral pulmonary artery stenosis (PPAS) is a rare condition seen as a cause of PH in adults.^89,90^ PH secondary to PPAS can be misdiagnosed as idiopathic pulmonary arterial hypertension or CTEPH.^89,90^ It can be seen as an isolated congenital anomaly in children or can occur as part of Noonan's, Alagille's, or William's syndromes.^89^ PPAS affects the peripheral smaller pulmonary arteries and can cause single or multiple stenoses in these locations.^89,91^

PPAS can present with clinical features of PH like exercise intolerance and dyspnea on exertion, and can be indistinguishable from patients with CTEPH. On physical exam, a continuous murmur is often heard along the sternal borders and anterior aspect of the chest depending on the location of the pulmonary stenosis.^91^ These flow murmurs have also been described in patients with CTEPH.^92^ Kreutzer et al.^93^ described 6 out of 12 patients with PPAS who were initially wrongly diagnosed as CTEPH. VQ scanning will reveal multiple segmental perfusion defects similar to CTEPH.^89,93^ Definitive diagnosis requires pulmonary angiography.^89,93^

Balloon pulmonary angioplasty (BPA) is the preferred treatment modality for PPAS although stenting is also used in adults sometimes.^89^ Relief of the obstruction with stenting or angioplasty can lead to reperfusion pulmonary edema due to sudden increase in pressure downstream of the stented vessel.^89^ In the series by Kreutzer,^93^ 9 out of 11 patients underwent successful BPA, 1 patient did not benefit from the procedure and 1 died due to pulmonary hemorrhage as a direct complication of BPA.^93^

## Summary

The diagnosis of CTEPH is challenging and critical at the same time, as dramatically effective procedures are available to the correctly diagnosed patient. The VQ scan remains the initial screening test of choice given its binary simple interpretation and high sensitivity for the detection of CTEPH. However, an abnormal VQ scan showing mismatched perfusion defects does not always equate to CTEPH. Furthermore, lining thrombus on CTA, typically reported as chronic pulmonary embolism, does not equate with CTEPH either. A multidisciplinary team with experience and expertise in CTEPH, pulmonary vascular disease, and diagnostic radiology is needed to arrive at the correct diagnosis and treatment plan.

## References

[1] Robbins IM Pugh ME Hemnes AR Update on chronic thromboembolic pulmonary hypertension. Trends Cardiovasc Med 2017; 27: 29–37. 27345156 10.1016/j.tcm.2016.05.010

[2] Pepke-Zaba J Ghofrani HA Hoeper MM Medical management of chronic thromboembolic pulmonary hypertension. Eur Respir Rev 2017; 26: 160107. 28356404 10.1183/16000617.0107-2016 PMC9488844

[3] Simonneau G Montani D Celermajer DS, et al. Haemodynamic definitions and updated clinical classification of pulmonary hypertension. Eur Respir J 2019; 53: 1801913. 30545968 10.1183/13993003.01913-2018 PMC6351336

[4] Lang IM Madani M Update on chronic thromboembolic pulmonary hypertension. Circulation 2014; 130: 508–518. 25092279 10.1161/CIRCULATIONAHA.114.009309

[5] McNeil K Dunning J Chronic thromboembolic pulmonary hypertension (CTEPH). Heart 2007; 93: 1152–1158. 17699182 10.1136/hrt.2004.053603 PMC1955041

[6] Olsson KM Meyer B Hinrichs J, et al. Chronic thromboembolic pulmonary hypertension. Dtsch Arztebl Int 2014; 111: 856–862. 25585582 10.3238/arztebl.2014.0856 PMC4357025

[7] Wolf M Boyer-Neumann C Parent F, et al. Thrombotic risk factors in pulmonary hypertension. Eur Respir J 2000; 15: 395–399. 10706510 10.1034/j.1399-3003.2000.15b28.x

[8] Gopalan D Delcroix M Held M Diagnosis of chronic thromboembolic pulmonary hypertension. Eur Respir Rev 2017; 26: 160108. 28298387 10.1183/16000617.0108-2016 PMC9488918

[9] McCann C Gopalan D Sheares K, et al. Imaging in pulmonary hypertension, part 2: large vessel diseases. Postgrad Med J 2012; 88: 317–325. 22282738 10.1136/postgradmedj-2011-130274

[10] Agarwal PP Wolfsohn AL Matzinger FR, et al. In situ central pulmonary artery thrombosis in primary pulmonary hypertension. Acta Radiol 2005; 46: 696–700. 16372688 10.1080/02841850500215501

[11] Gopalan D McCann C Sheares K, et al. Imaging in pulmonary hypertension, part 3: small vessel diseases. Postgrad Med J 2012; 88: 397–406. 22267525 10.1136/postgradmedj-2011-130276

[12] Lee C-W Huang S-S Huang P-H Pulmonary arterial thrombosis in a patient with an atrial septal defect and Eisenmenger syndrome. Korean Circ J 2012; 42: 772–775. 23236330 10.4070/kcj.2012.42.11.772 PMC3518712

[13] Peña E Dennie C Veinot J, et al. Pulmonary hypertension: how the radiologist can help. Radiographics 2012; 32: 9–32. 22236891 10.1148/rg.321105232

[14] Maeda K Saiki Y Yamaki S In situ thrombosis of small pulmonary arteries in pulmonary hypertension developing after chemotherapy for malignancy. Pulm Med 2015; 2015: 230846. 25692040 10.1155/2015/230846 PMC4322293

[15] Silversides CK Granton JT Konen E, et al. Pulmonary thrombosis in adults with Eisenmenger syndrome. J Am Coll Cardiol 2003; 42: 1982–1987. 14662263 10.1016/j.jacc.2003.07.022

[16] Bergin CJ Rios G King MA, et al. Accuracy of high-resolution CT in identifying chronic pulmonary thromboembolic disease. Am J Roentgenol 1996; 166: 1371–1377. 8633450 10.2214/ajr.166.6.8633450

[17] Levine D Al-Naamani N Channick R, et al. Pulmonary artery filling defects: are they all the same?. Adv Pulm Hypertens 2014; 13: 122–124.

[18] Memon HA Lin CH Guha A Chronic thromboembolic pulmonary hypertension: pearls and pitfalls of diagnosis. Method DeBakey Cardiovasc J 2016; 12: 199–204. 10.14797/mdcj-12-4-199 PMC5344469 28289494

[19] Bandyopadhyay D Panchabhai TS Bajaj NS, et al. Primary pulmonary artery sarcoma: a close associate of pulmonary embolism-20-year observational analysis. J Thorac Dis 2016; 8: 2592–2601. 27747013 10.21037/jtd.2016.08.89 PMC5059338

[20] Chong S Kim TS Kim BT, et al. Pulmonary artery sarcoma mimicking pulmonary thromboembolism: integrated FDG PET/CT. AJR Am J Roentgenol 2007; 188: 1691–1693. 17515395 10.2214/AJR.05.0874

[21] Huo L Moran CA Fuller GN, et al. Pulmonary artery sarcoma: a clinicopathologic and immunohistochemical study of 12 cases. Am J Clin Pathol 2006; 125: 419–424. 16613346

[22] Yi CA Lee KS Choe YH, et al. Computed tomography in pulmonary artery sarcoma: distinguishing features from pulmonary embolic disease. J Comput Assist Tomogr 2004; 28: 34–39. 14716229 10.1097/00004728-200401000-00005

[23] Wong HH Gounaris I McCormack A, et al. Presentation and management of pulmonary artery sarcoma. Clin Sarcoma Res 2015; 5: 3. 25628857 10.1186/s13569-014-0019-2 PMC4307142

[24] Kim C Kim MY Kang JW, et al. Pulmonary artery intimal sarcoma versus pulmonary artery thromboembolism: CT and clinical findings. Korean J Radiol 2018; 19: 792–802. 29962886 10.3348/kjr.2018.19.4.792 PMC6005959

[25] Liu X Hou J Wang X, et al. An intimal sarcoma of pulmonary artery mimicking pulmonary embolism: a case report and literature review. Respirol Case Rep 2017; 5: e00248. 28674613 10.1002/rcr2.248 PMC5488381

[26] Renapurkar R Shrikanthan S Heresi G, et al. Imaging in chronic thromboembolic pulmonary hypertension. J Thoracic Imag 2017; 32: 71–88. 10.1097/RTI.0000000000000256 28060193

[27] Bressler EL Nelson JM Primary pulmonary artery sarcoma: diagnosis with CT, MR imaging, and transthoracic needle biopsy. AJR Am J Roentgenol 1992; 159: 702–704. 1529830 10.2214/ajr.159.4.1529830

[28] Khan N Jawad A Cicenia J, et al. Use of EBUS-TBNA in the diagnosis of primary pulmonary artery sarcoma. Am J Respir Crit Care Med 2017; 195: A6650.

[29] Dartevelle P Fadel E Mussot S, et al. Chronic thromboembolic pulmonary hypertension. Eur Respir J 2004; 23: 637–648. 15083767 10.1183/09031936.04.00079704

[30] Hu Y Qiu JX Liao JP, et al. Clinical manifestations of fibrosing mediastinitis in Chinese patients. Chin Med J 2016; 129: 2697–2702. 27824002 10.4103/0366-6999.193457 PMC5126161

[31] McNeeley MF Chung JH Bhalla S, et al. Imaging of granulomatous fibrosing mediastinitis. AJR Am J Roentgenol 2012; 199: 319–327. 22826392 10.2214/AJR.11.7319

[32] Li Y Meng X Wang Y, et al. Fibrosing mediastinitis with pulmonary hypertension as a complication of pulmonary vein stenosis: a case report and review of the literature. Medicine 2018; 97: e9694. 29369193 10.1097/MD.0000000000009694 PMC5794377

[33] Rossi SE McAdams HP Rosado-de-Christenson ML, et al. Fibrosing mediastinitis. Radiographics 2001; 21: 737–757. 11353121 10.1148/radiographics.21.3.g01ma17737

[34] Wu Z Jarvis H Howard LS, et al. Post-tuberculous fibrosing mediastinitis: a review of the literature. BMJ Open Respir Res 2017; 4: e000174. 10.1136/bmjresp-2016-000174 PMC5501238 28725444

[35] Seferian A Steriade A Jaïs X, et al. Pulmonary hypertension complicating fibrosing mediastinitis. Medicine 2015; 94: e1800. 26554778 10.1097/MD.0000000000001800 PMC4915879

[36] Kalantari KR Malek H Amin A, et al. Chronic thromboembolic pulmonary hypertension versus fibrosing mediastinitis. Anatol J Cardiol 2019; 21: E4–E5. 10.14744/AnatolJCardiol.2018.12258 PMC6457413 30694812

[37] Kuranga AO Eubank AM Bowling MR Fibrosing mediastinitis: a review of epidemiology, diagnosis and management. Int J Respir Pulm Med 2018; 5: 79.

[38] Bourlier D O'Connell C Montani D, et al. A rare case of sarcoidosis-associated pulmonary hypertension in a patient exposed to silica. Eur Respir Rev 2016; 25: 93–96. 26929426 10.1183/16000617.0073-2015 PMC9487671

[39] Ostwani W Arabi M Alteration in pulmonary perfusion due to iatrogenic pulmonary vein stenosis: a mimicker of pulmonary embolism. Avicenna J Med 2011; 1: 58–60. 23210011 10.4103/2231-0770.90918 PMC3507064

[40] Pazos-López P García-Rodríguez C Guitián-González A, et al. Pulmonary vein stenosis: etiology, diagnosis and management. World J Cardiol 2016; 8: 81–88. 26839659 10.4330/wjc.v8.i1.81 PMC4728109

[41] Saad EB Marrouche NF Saad CP, et al. Pulmonary vein stenosis after catheter ablation of atrial fibrillation: emergence of a new clinical syndrome. Ann Intern Med 2003; 138: 634–638. 12693885 10.7326/0003-4819-138-8-200304150-00010

[42] Fender EA Widmer RJ Hodge DO, et al. Severe pulmonary vein stenosis resulting from ablation for atrial fibrillation: presentation, management, and clinical outcomes. Circulation 2017; 135: e1016. 28461424 10.1161/CIRCULATIONAHA.117.027480

[43] Wang W Zhou JP Wu LQ, et al. Pulmonary-vein stenosis can mimic massive pulmonary embolism after radiofrequency ablation for atrial fibrillation. Respir Care 2011; 56: 874–877. 21333083 10.4187/respcare.00975

[44] Galizia M Renapurkar R Prieto L, et al. Radiologic review of acquired pulmonary vein stenosis in adults. Cardiovasc Diagn Ther 2018; 8: 387–398. 30057885 10.21037/cdt.2018.05.05 PMC6039802

[45] Kumar N Aksoy I Pison L, et al. Management of pulmonary vein stenosis following catheter ablation of atrial fibrillation. J Atr Fibrillat 2014; 7: 1060. 10.4022/jafib.1060 PMC5135150 27957081

[46] Di Biase L Fahmy TS Wazni OM, et al. Pulmonary vein total occlusion following catheter ablation for atrial fibrillation: clinical implications after long-term follow-up. J Am Coll Cardiol 2006; 48: 2493–2499. 17174188 10.1016/j.jacc.2006.08.038

[47] Grosse C Grosse A CT findings in diseases associated with pulmonary hypertension: a current review. Radiographics 2010; 30: 1753–1777. 21057119 10.1148/rg.307105710

[48] Bailey CL Channick RN Auger WR, et al. “High probability” perfusion lung scans in pulmonary venoocclusive disease. Am J Respir Crit Care Med 2000; 162: 1974–1978. 11069842 10.1164/ajrccm.162.5.2003045

[49] Mandel J Mark EJ Hales CA Pulmonary veno-occlusive disease. Am J Respir Crit Care Med 2000; 162: 1964–1973. 11069841 10.1164/ajrccm.162.5.9912045

[50] Seferian A Helal B Jaïs X, et al. Ventilation/perfusion lung scan in pulmonary veno-occlusive disease. Eur Respir J 2012; 40: 75–83. 22088969 10.1183/09031936.00097911

[51] Montani D Achouh L Dorfmüller P, et al. Pulmonary veno-occlusive disease: clinical, functional, radiologic, and hemodynamic characteristics and outcome of 24 cases confirmed by histology. Medicine 2008; 87: 220–233. 18626305 10.1097/MD.0b013e31818193bb

[52] Dion J Terrier B Jaïs X, et al. Atypical vasculitis mimicking chronic thromboembolic pulmonary hypertension. Am J Med 2015; 128: e47–49. 10.1016/j.amjmed.2015.05.028 26071824

[53] Hagan G Gopalan D Church C, et al. Isolated large vessel pulmonary vasculitis as a cause of chronic obstruction of the pulmonary arteries. Pulm Circ 2011; 1: 425–429. 22140633 10.4103/2045-8932.87312 PMC3224435

[54] Yamada I Shibuya H Matsubara O, et al. Pulmonary artery disease in Takayasu's arteritis: angiographic findings. AJR Am J Roentgenol 1992; 159: 263–269. 1352939 10.2214/ajr.159.2.1352939

[55] Zhang Yu-Hui Song Wei-Min, et al. Initial isolated Takayasu's arteritis of bilateral pulmonary artery branches. Rev Brasil Reumatol 2017; 57: 626–629. 10.1016/j.rbre.2016.02.002 29173701

[56] Toledano K Guralnik L Lorber A, et al. Pulmonary arteries involvement in Takayasu's arteritis: two cases and literature review. Semin Arthritis Rheum 2011; 41: 461–470. 21803399 10.1016/j.semarthrit.2011.06.001

[57] Fukuda Y Shirai K Takamiya Y, et al. Isolated pulmonary arterial stenosis caused by Takayasu's arteritis in an elderly male. J Cardiol 2008; 51: 196–200. 18522795 10.1016/j.jjcc.2007.12.003

[58] Nakajima N Takayasu arteritis: consideration of pulmonary involvement. Ann Vasc Dis 2008; 1: 7–10. 23555332 10.3400/avd.AVDedit00107 PMC3610226

[59] Kerr KM Auger WR Fedullo PF, et al. Large vessel pulmonary arteritis mimicking chronic thromboembolic disease. Am J Respir Crit Care Med 1995; 152: 367–373. 7599847 10.1164/ajrccm.152.1.7599847

[60] Wang X Dang A Chen B, et al. Takayasu arteritis-associated pulmonary hypertension. J Rheumatol 2015; 42: 495–503. 25593224 10.3899/jrheum.140436

[61] Mahmoud S Ghosh S Farver C, et al. Pulmonary vasculitis: spectrum of imaging appearances. Radiol Clin North Am 2016; 54: 1097–1118. 27719978 10.1016/j.rcl.2016.05.007

[62] Zhu FP Luo S Wang ZJ, et al. Takayasu arteritis: imaging spectrum at multidetector CT angiography. Br J Radiol 2012; 85: e1282–e1292. 23175494 10.1259/bjr/25536451 PMC3611735

[63] Tezuka D Haraguchi G Ishihara T, et al. Role of FDG PET-CT in Takayasu arteritis: sensitive detection of recurrences. JACC Cardiovasc Imag 2012; 5: 422–429. 10.1016/j.jcmg.2012.01.013 22498333

[64] Dhagat PK Singh S Jain M, et al. Thoracic sarcoidosis: imaging with high resolution computed tomography. J Clin Diagn Res 2017; 11: TC15–TC18. 10.7860/JCDR/2017/24165.9459 PMC5376893 28384959

[65] Montani D Günther S Dorfmüller P, et al. Pulmonary arterial hypertension. Orphanet J Rare Dis 2013; 8: 97. 23829793 10.1186/1750-1172-8-97 PMC3750932

[66] Bazmpani MA Arsos G Zarogoulidis P, et al. A case of sarcoidosis-associated pulmonary hypertension masquerading as chronic thromboembolic pulmonary hypertension. Pulm Circ 2018; 8(3): 2045894018768289. 29537341 10.1177/2045894018768289 PMC5950934

[67] Tandon R Baughman RP Stanley J, et al. The link between chronic thromboembolic pulmochronic thromboembolic pulmonary hypertension (CTEPH) and sarcoidosis are recognized causes of nary hypertension and sarcoidosis: association or visual masquerade?. Sarcoidosis Vasc Diffuse Lung Dis 2017; 34: 352–355. 10.36141/svdld.v34i4.5852 PMC7170075 32476868

[68] Diaz-Guzman E Farver C Parambil J, et al. Pulmonary hypertension caused by sarcoidosis. Clin Chest Med 2008; 29: 549. 18539244 10.1016/j.ccm.2008.03.010 PMC2593121

[69] Huitema MP Grutters JC Rensing BJWM, et al. Pulmonary hypertension complicating pulmonary sarcoidosis. Neth Heart J 2016; 24: 390–399. 27194118 10.1007/s12471-016-0847-1 PMC4887307

[70] Godbole R Saggar R Zider A, et al. Insights on pulmonary tumor thrombotic microangiopathy: a seven-patient case series. Pulm Circ 2017; 7: 813–820. 28782988 10.1177/2045893217728072 PMC5703123

[71] RH Winterbauer IB Elfenbein WC Ball Jr Incidence and clinical significance of tumor embolization to the lungs. Am J Med 1968; 45: 271–290. 4299069 10.1016/0002-9343(68)90044-2

[72] Rokadia HK Heresi GA Tan CD, et al. A 33-year-old man with multiple bilateral pulmonary pseudoaneurysms. Chest 2015; 148: e112–e117. 26437818 10.1378/chest.15-0624

[73] Castañer E Gallardo X Rimola J, et al. Congenital and acquired pulmonary artery anomalies in the adult: radiologic overview. Radiographics 2006; 26: 349–371. 16549603 10.1148/rg.262055092

[74] Kridel R Myit S Pache JC, et al. Pulmonary tumor embolism: a rare cause of acute right heart failure with elevated D-dimers. J Thorac Oncol 2008; 3: 1482–1483. 19057276 10.1097/JTO.0b013e31818e107c

[75] Aiyappan V Alwail A Pulmonary tumor thromboembolism: a case report and review of literature. Ann Thorac Med 2007; 2: 169–170. 19727370 10.4103/1817-1737.36553 PMC2732100

[76] Roberts KE Hamele-Bena D Saqi A, et al. Pulmonary tumor embolism: a review of the literature. Am J Med 2003; 115: 228–232. 12935829 10.1016/s0002-9343(03)00305-x

[77] Suffredini DA Lee JM Peer CJ, et al. Pulmonary tumor thrombotic microangiopathy and pulmonary veno-occlusive disease in a woman with cervical cancer treated with cediranib and durvalumab. BMC Pulm Med 2018; 18: 112. 29996818 10.1186/s12890-018-0681-x PMC6042377

[78] Price LC Seckl MJ Dorfmüller P, et al. Tumoral pulmonary hypertension. Eur Respir Rev 2019; 28(151): pii 180065. 30728162 10.1183/16000617.0065-2018 PMC9489033

[79] Bonderman D Wilkens H Wakounig S, et al. Risk factors for chronic thromboembolic pulmonary hypertension. Eur Respir J 2009; 33: 325–331. 18799507 10.1183/09031936.00087608

[80] Carter BW Lichtenberger JP 3rd Wu CC Congenital abnormalities of the pulmonary arteries in adults. AJR Am J Roentgenol 2014; 202: W308–W313. 24660728 10.2214/AJR.13.11759

[81] Liu B Monroe EJ Kogut MJ Proximal interruption of the pulmonary artery: transcatheter embolization for emergent management of massive hemoptysis. Radiol Case Rep 2013; 8: 865. 27330642 10.2484/rcr.v8i3.865 PMC4900122

[82] Castañer E Gallardo X Ballesteros E, et al. CT diagnosis of chronic pulmonary thromboembolism. Radiographics 2009; 29: 31–50. discussion 50–53. 19168835 10.1148/rg.291085061

[83] Anand SH Jasper A Mani SE, et al. Proximal interruption of the pulmonary artery: a case series. J Clin Diagnos Res 2015; 9: TD04–TD06. 10.7860/JCDR/2015/16198.6980 PMC4717800 26816968

[84] Moser KM Olson LK Schlusselberg M, et al. Chronic thromboembolic occlusion in the adult can mimic pulmonary artery agenesis. Chest 1989; 95: 503–508. 2920575 10.1378/chest.95.3.503

[85] Muthusami P Ananthakrishnan R Elangovan S Incidentally detected unilateral pulmonary artery agenesis with pulmonary hypoplasia in a 67 year old woman. J Radiol Case Rep 2010; 4: 32–37. 10.3941/jrcr.v4i11.583 PMC3303358 22470700

[86] Singhi AK Francis E Kumar RK Isolated absence of right pulmonary artery. Ann Pediatr Cardiol 2010; 3: 119–122. 21234189 10.4103/0974-2069.74037 PMC3017914

[87] Chen IC Chen YW Lin SH, et al. Usefulness of combination of pulmonary ventilation and perfusion scintigraphy on the diagnosis of children with unilateral hyperlucent lung. Nucl Med Commun 2011; 32: 1052–1059. 21869729 10.1097/MNM.0b013e32834a6dfd

[88] Chun JY Morgan R Belli AM Radiological management of hemoptysis: a comprehensive review of diagnostic imaging and bronchial arterial embolization. Cardiovasc Intervent Radiol 2010; 33: 240–250. 20058006 10.1007/s00270-009-9788-z

[89] Kushner T Halperin JL Nair AP, et al. Peripheral pulmonary artery stenosis masquerading as pulmonary hypertension: a diagnostic and therapeutic challenge. Vasc Med 2012; 17: 235–238. 22848086 10.1177/1358863X12451207

[90] Tonelli AR Ahmed M Hamed F, et al. Peripheral pulmonary artery stenosis as a cause of pulmonary hypertension in adults. Pulm Circ 2015; 5: 204–210. 25992283 10.1086/679727 PMC4405719

[91] Baum D Khoury GH Ongley PA, et al. Congenital stenosis of the pulmonary artery branches. Circulation 1964; 29: 680–687. 14156862 10.1161/01.cir.29.5.680

[92] Auger WR Kerr KM Kim NH, et al. Evaluation of patients with chronic thromboembolic pulmonary hypertension for pulmonary endarterectomy. Pulm Circ 2012; 2: 155–162. 22837856 10.4103/2045-8932.97594 PMC3401869

[93] Kreutzer J Landzberg MJ Preminger TJ, et al. Isolated peripheral pulmonary stenoses in the adult. Circulation 1996; 93: 1417–1423. 8641032 10.1161/01.cir.93.7.1417

